# Population Pharmacokinetic Modeling of Certepetide in Human Subjects With Metastatic Pancreatic Ductal Adenocarcinoma

**DOI:** 10.1002/cpdd.1502

**Published:** 2025-01-09

**Authors:** Alex Winning, William K. Sietsema, Kristen K. Buck, Abigail Linsmeier, Pawel Wiczling

**Affiliations:** ^1^ Department of Pharmacometrics Modeling A2‐Ai LLC Ann Arbor MI USA; ^2^ Research and Development Lisata Therapeutics, Inc. Basking Ridge NJ USA; ^3^ Department of Biopharmaceutics and Pharmacodynamics Medical University of Gdańsk Gdańsk Poland

**Keywords:** certepetide, clinical research, first‐in‐human, modeling and simulation, oncology, population pharmacokinetics

## Abstract

Certepetide (aka LSTA1 and CEND‐1) is a novel cyclic tumor‐targeting internalizing arginyl glycylaspartic acid peptide being developed to treat solid tumors. Certepetide is designed to overcome existing challenges in treating solid tumors by delivering co‐administered anticancer drugs into the tumor while selectively depleting immunosuppressive T cells, enhancing cytotoxic T cells in the tumor microenvironment, and inhibiting the metastatic cascade. A population pharmacokinetic (PK) analysis was conducted to characterize the concentration‐time profile of patients with metastatic exocrine pancreatic cancer receiving certepetide in combination with nab‐paclitaxel and gemcitabine, and to investigate the effects of clinically relevant covariates on PK parameters. The PK of certepetide was characterized by a 2‐compartment model with linear elimination and a proportional residual error structure. Body weight and baseline creatinine clearance (CrCL) were found to have statistically significant effects on central and peripheral volume (Vc and Vp) and clearance (CL) parameters, respectively, during model development and were included as covariate effects in the final PK model. Forest plots demonstrated a potentially clinically meaningful impact of high body weight (100 kg) on certepetide exposure (steady‐state maximum concentration [C_max,ss_] and area under the concentration‐time curve [AUC_ss_]), as well as low and high CrCL (50 and 150 mL/min) on AUC_ss_. Exposure predictions illustrated a relationship between certepetide exposure (AUC_ss_) and renal function, with increasing exposure and decreasing CL of certepetide observed with worsening renal function. Modeling will strengthen the understanding of certepetide's PKs and will inform dose optimization in ongoing drug development activities.

It is well recognized that treatment for solid tumors is suboptimal and there is an urgent need for novel therapies. Treatment of solid tumors remains a significant challenge for 2 main reasons, solid tumors are surrounded by a dense cellular barrier called the stroma which inhibits anticancer agents from effectively penetrating the tumor and solid tumors have an immunosuppressive tumor microenvironment that hinders the immune system from recognizing and attacking the cancer.^1^ Unfortunately, prolonged and/or escalated conventional anticancer therapies do not overcome these challenges and often lead to intolerable off‐target side effects.

Pancreatic ductal adenocarcinoma (PDAC) is a solid tumor with a hostile tumor microenvironment and abundant stroma that compromises drug delivery and may explain the significant morbidity and mortality therein.^2^ By 2030, it is believed that pancreatic cancer will have become the second leading cause of cancer‐related death in the United States.^3^ Despite the advances in knowledge regarding tumor biology and treatment of PDAC, the prognosis for these patients remains poor, with a 5‐year survival rate of only 12%.^4^

PDAC is often associated with elevated levels of CA19‐9, a tumor biomarker measured in the serum. CA19‐9 levels often correlate with the stage of disease, with higher levels of CA19‐9 more commonly seen in advanced stages of PDAC. Once diagnosed with pancreatic cancer, regular CA 19‐9 tests can be used to monitor treatment response and detect potential recurrence.^5^

Certepetide (aka LSTA1 and CEND‐1) is a novel 9‐amino acid cyclic tumor‐targeting internalizing arginyl glycylaspartic acid peptide (molecular weight 989.1 g/mol) designed to overcome the challenges in treating solid tumors. Certepetide selectively targets and enhances the penetration of coadministered anticancer drugs and modifies the tumor microenvironment.^6^, ^7^, ^8^ Specifically, the arginyl glycylaspartic acid motif of certepetide binds to αvβ3/5‐integrins which are upregulated on tumor endothelium.^6^ On binding and subsequent proteolytic cleavage, the resulting 5 amino‐acid linear C‐end‐Rule peptide binds to neuropilin‐1, which is also overexpressed on tumor vascular endothelial cells and tumor cells.^9^ This process triggers an active transport mechanism to deliver anticancer drugs into the tumor.^8^, ^10^, ^11^ Due to the nature of the transport mechanism, penetration is enhanced for small molecules,^12^ monoclonal antibodies,^10^, ^13^ and larger moieties such as nanoparticles^14^ and cell therapies.^9^, ^15^ Certepetide also selectively depletes immunosuppressive T cells, enhances cytotoxic T cells, and inhibits the metastatic cascade.^16^, ^17^, ^18^

Certepetide is administered as a slow intravenous push over 1 minute shortly after the administration of chemotherapy and/or immunotherapy. Certepetide is thought to be metabolized into its component amino acids, as expected for small peptides. Autoradiography has demonstrated that certepetide primarily undergoes renal elimination (data on file). Certepetide does not bind to plasma proteins (data on file).

Preliminary analysis of the safety, tolerability, PK, and biological activity of certepetide including 31 patients with unresectable metastatic PDAC receiving first‐line treatment with certepetide in combination with nab‐paclitaxel and gemcitabine has already been published.^19^, ^20^ Investigators and associated Human Research Ethics Committees are provided in Table S1. The majority of patients were male (65%) and Caucasian (87%). No patients had any prior cancer medication, radiation, or pancreatic cancer surgery and 84% of patients had elevated cancer antigen 19‐9 (CA19‐9). The median age of all patients was 62 years.

Certepetide exposure concentrations were observed to be consistent throughout the course of the study. The mean baseline CA19‐9 concentration was 23,469 U/mL. Nearly all patients (20 out of 22) had a ≥50% reduction in CA19‐9 at cycle 5, day 1.

All 31 patients in this phase 1 trial had ≥1 treatment‐emergent adverse event, 94% had adverse events (AEs) of severity grade 3 or 4, and 71% had serious adverse advents. The most common AEs observed were neutropenia, anemia, leukopenia, and pulmonary embolism. The 3.2 and 1.6 mg/kg dose groups were comparable in the number and severity of AEs observed. There were no dose‐limiting toxicities associated with the study drug. The adverse event profile of patients in this study was comparable to that of subjects with pancreatic cancer treated with gemcitabine and nab‐paclitaxel. A total of 10 deaths occurred during the study period: 9 deaths were due to metastatic pancreatic cancer disease progression and 1 death was due to left middle cerebral artery stroke due to disease progression (not attributed to certepetide).

Preliminary efficacy results were summarized as follows: investigator‐assessed overall response rate was 59%, disease control rate at 16 weeks was 90%, 50% of patients in the 1.6 mg/kg dose group and 61.5% of patients in the 3.2 mg/kg dose group had a partial response or complete response, median progression‐free survival after 33.2 months was 9.7 months, median overall survival after 26 months was 13.2 months, and median overall survival from treatment start was 12.8 months.

In this work, the population pharmacokinetics (PKs) of certepetide in a first‐in‐human trial involving patients with metastatic exocrine pancreatic cancer was investigated. Additionally, potential nonlinearity in certepetide PK was examined and the influence of patient‐specific factors (age, body weight, sex, and CrCL) on its PKs was evaluated.

## Methods

### Clinical Study Methods

The clinical aspects of this study were conducted in accordance with the principles of the Declaration of Helsinki and the National Health and Medical Research Council of Australia's National Statement on Ethical Conduct in Human Research 2018. The research and accompanying materials were approved by applicable Human Research Ethics Committees as previously described in the paper by Dean et al^19^ and outlined in Table S1. The study was registered on the Clinical Trials registry under the identifier: NCT03517176.

The clinical aspects of this study were conducted at 3 clinical sites in Australia. All subjects gave their informed consent. The study enrolled subjects with metastatic PDAC and was a rising dose design, with certepetide doses of 0.2, 0.8, 1.6, and 3.2 mg/kg body weight. Each subject received a certepetide monotherapy run‐in followed by 28‐day cycles of certepetide with standard‐of‐care chemotherapy (nab‐paclitaxel and gemcitabine), as previously described.^19^ Each 28‐day cycle involved dosing with nab‐paclitaxel, gemcitabine, and certepetide on days 1, 8, and 15. In each dosing instance, nab‐paclitaxel was given first intravenously at a dose of 125 mg/m^2^ over 30 minutes. This was immediately followed by the administration of certepetide as a slow intravenous push over 1 minute. Gemcitabine was given as an intravenous infusion at a dose of 1000 mg/m^2^ over 30 minutes. Samples for PK analysis were collected before certepetide dosing and at 3, 15, and 30 minutes and 1, 3, and 6 hours after completion of the certepetide infusion. PK collections were performed during a monotherapy run‐in period and on day 1 of chemotherapy cycles 1 and 6. Modeling was performed with all available data.

### Bioanalytical Assay

Certepetide concentrations in human plasma were determined using a liquid chromatography‐tandem mass spectrometry method across a concentration range of 50.0‐2500 ng/mL, with 50.0 ng/mL as the lower limit of quantification. Briefly, a plasma sample was spiked with a deuterated internal standard and then precipitated by methanol. After centrifugation, the supernatant was transferred to a clean tube and evaporated under nitrogen. Following reconstitution with 5% methanol:water, the sample was injected into a Shimadzu Nexera UPLC that used a Kinetex EVO‐C18 column for separation using first mobile phase A (0.1% formic acid in deionized water) followed by mobile phase B (0.1% formic acid in methanol). The multiple reaction monitoring transition was monitored on an ABSciex QTRAP 5500 mass spectrometer. The transitions used for certepetide and deuterated certepetide (+6) were 494.9 → 70.1 and 497.7 → 75.0, respectively. For intraday runs, the average accuracy and precision were 10.2% and 9.6%, respectively, at a concentration of 150 ng/mL. The inter‐day accuracy and precision were 10.2% and 9.7%, respectively.

### Analysis Dataset

Source data included PK concentrations, sample dates and times, dose amounts and times, subject demographics, and other relevant covariates in SAS format (SAS7BDAT, XPT). Derived datasets were prepared for nonlinear mixed‐effects modeling (NONMEM) using R (Version 4.3.1).^21^

The PK analysis dataset was composed of actual dates and times of dose and concentration samples. Post‐dose PK concentration data below the limit of quantification were accounted for using the M1 method (ie, excluded from the analysis).^22^ Subject covariates at baseline were derived from the source data at the baseline visit, the screening visit, or another visit before the start of certepetide administration.

### Software

NONMEM software (version 7.5.1, ICON Development Solutions)^23^ was used to perform nonlinear mixed‐effects modeling. Data assembly, graphical analyses, and model simulations were performed using NONMEM (version 7.5.1) and R (version 4.3.1).

### Pharmacokinetic Model Development

Concentration and dose‐normalized concentration‐time profiles, stratified by dose level, were generated to provide insight into appropriate model structures for initial model development.

A 2‐compartment model with linear elimination was used as the starting point for model development. Interindividual variability was included in structural model parameters (eg, central volume of distribution [Vc], clearance [CL], peripheral volume of distribution [Vp], and distribution clearance [Q]). Interindividual variability, assumed to have log‐normal distribution, was calculated using Equation (1).

(1) $$\;\theta_{P,i}=\theta_{P,\mathrm{typical}}\;\cdot e^{\eta_{P,i}}$$

The individual variables in Equation (1) are defined as follows:

- $\theta_{P,i}$ is the individual value of the PK parameter P for the ith subject.
- $\theta_{P,\mathrm{typical}}$ is the NONMEM‐determined typical value of the PK parameter P in a population.
- $\eta_{P,i}$ is the interindividual random effect accounting for the ith subject's deviation from the typical value for parameter P.$\;\eta_{P,i}$ is assumed to be normally distributed, with a mean of 0 and a variance of $\omega_{P}^{2}$.

The percentage coefficient of variation (CV) for interindividual variability (IIV) was calculated using Equation (2).

(2) $$\%CV_{P}\;\left(\mathrm{IIV}\right)=\sqrt{\left(e^{\left(\omega_{P}^{2}\right)}-1\right)}\;\times 100$$

A diagonal variance‐covariance matrix was estimated for the vector of interindividual random effects. The shrinkage of individual random effects was calculated using Equation (3). In this equation, $SD_{P}$ is defined as the standard deviation of the $\eta_{P}$ and $\Omega_{\left(P,P\right)}$ is defined as the corresponding diagonal term of the variance‐covariance matrix.

(3) $$\mathrm{Shrinkag}e_{P}=100\times \left(1-\frac{SD_{P}}{\sqrt{\Omega_{\left(P,P\right)}}}\right)$$

The residual variance was initially modeled using an additive and proportional error model (Equation 4), which accounted for variability between subjects, assay error, model misspecification, and any potential data issues (eg, incorrect dose or sample collection times).

(4) $$\;Y_{\mathrm{ij}}=\mathrm{IPRE}D_{\mathrm{ij}}\times \left(1+\varepsilon_{1,\mathrm{ij}}\right)+\varepsilon_{2,\mathrm{ij}}$$

The individual variables in Equation (4) are defined as follows:

- $Y_{\mathrm{ij}}$ is the observed concentration for the ith subject at time j.
- $\mathrm{IPRE}D_{\mathrm{ij}}$ is the individual predicted concentration based on the model.
- $\varepsilon_{1,\mathrm{ij}}$ is the constant coefficient of the variation error term and $\varepsilon_{2,\mathrm{ij}}$ is the additive error term. Both residual error terms are from independent normal distributions with a mean of 0 and variance $\sigma_{1}^{2}$ and $\sigma_{2}^{2}$.

Other residual error models (eg, additive or proportional) were tested during the model development process.

Equation (5) was used to calculate the standard deviation of the residual error (W).

(5) $$\;W_{\mathrm{ij}}=\sqrt{\sigma_{1}^{2}\times {\mathrm{IPRED}}_{\mathrm{ij}}^{2}+\sigma_{2}^{2}}\;$$

Model development continued by adding covariates in a stepwise fashion and assessing the change in objective function value (OFV). Potential covariates were selected based on clinical interest and included the following: body weight, age, sex on all PK parameters, and baseline creatinine clearance (CrCL) calculated using the Cockcroft–Gault formula on CL. Covariates which resulted in a large change in OFV were retained in the PK model. A backward elimination procedure was then performed to select the most parsimonious model. During this procedure, covariate effect parameters were removed from the model and the resulting model was evaluated based on the following significance criteria: *α* = 0.001 (ΔOFV = 10.8). If the significance criteria were not met, the covariate parameter effects were removed from the model.

A power model was used to describe the relationship between continuous covariates of age and body weight, and the typical value of PK parameters, as described by Equation (6).

(6) $$\;\theta_{P,i}=\theta_{P,\mathrm{typical}}\;\times {\left(\frac{x_{i}}{x_{\mathrm{ref}}}\right)}^{\theta_{P,x}}$$

The individual variables in Equation (6) are defined as follows:

- $\theta_{P,i}$ is the individual value of the PK parameter P for the ith subject; $x_{i}$ is the value of covariate x for subject i.
- $\theta_{P,\mathrm{typical}}$ and $\theta_{P,x}$ are the fixed‐effect parameters.
- $x_{\mathrm{ref}}$ is a reference value of the covariate x. The reference value was either a population median or a standard reference value (ie, 60 years of age, 70 kg body weight).

A linear model was used to describe the relationship between continuous covariate CrCL and the typical value of CL, as described by Equation (7).

(7) $$\theta_{\mathrm{CL},i}=\theta_{\mathrm{CL},\mathrm{NR}}+\theta_{\mathrm{CL},R}\times \frac{\mathrm{BCRC}L_{i}}{90}$$

The individual variables in Equation (7) are defined as follows:

- $\theta_{\mathrm{CL},i}$ is the individual value of CL for the ith subject.
- $\theta_{\mathrm{CL},\mathrm{NR}}$ is the nonrenal (independent of CrCL) clearance.
- $\theta_{\mathrm{CL},R}$ is the renal (dependent on CrCL) clearance.
- $\mathrm{BCRC}L_{i}$ is baseline CrCL for subject i and 90 mL/min refers to the reference value for CrCL derived via the Cockcroft–Gault formula.

A proportional model was used to describe the relationship between categorical covariates and the typical value of PK parameters, as described in Equation (8).

(8) $$\;\theta_{P,i}=\theta_{P,\mathrm{typical}}\;\cdot \left(1+\theta_{P,x}\times x_{i}\right)$$

The individual variables in Equation (8) are defined as follows:

- $\theta_{P,i}$ is the individual value of the PK parameter P for the ith subject.
- $\theta_{P,\mathrm{typical}}$ and $\theta_{P,x}$ are the fixed‐effect parameters. The lower boundary for $\theta_{P,x}$ was set equal to −1 to ensure a positive value of PK parameter estimates.
- $x_{i}$ is the indicator variable dependent on the covariate category for a subject i.

### Model Validation

Throughout the model development process, the model was assessed for adequate model fit and predictive ability using various diagnostic procedures and visual predictive checks (VPCs).

Both numerical and graphical diagnostic procedures were employed to evaluate the model development process, following guidance in the US Food and Drug Administration Population Pharmacokinetics Guidance Document. Specifically, the following factors were taken into consideration: minimization and completion of the covariance step in NONMEM, reduction in OFV and residual variance, model stability indicated by condition number <1000,^24^ and observed alignment between observed and model‐predicted concentrations in standard goodness‐of‐fit plots.^25^

Internal VPCs were used to ensure the predictive ability of the final PK model.^26^ To generate the VPCs, 500 datasets were simulated using PK parameter estimates fixed to final values from the PK model to reproduce key components of the observed dataset, including design, dosing regimens, number of subjects, and proportions of covariates. From these simulated datasets, the 5th, 50th, and 95th percentiles, with 95% nonparametric confidence intervals, were identified for each replicate within a time bin and compared to percentiles identified from the observed data across time bins. The criterion for declaring a final PK model is the containment of the observed percentiles within the simulated confidence intervals.

### Model Applications

Forest plots were generated to assess the potential clinical impact of covariate effects on certepetide exposure (ie, steady‐state maximum concentration [C_max,ss_] and area under the concentration–time curve [AUC_ss_]) following a dosing regimen of 3.2 mg/kg every 8 hours for a reference 70‐kg subject. To produce the forest plots, a smoothed parametric bootstrap procedure was used to create 1000 sets of parameter estimates. The smoothed parametric bootstrap procedure uses the vector of population parameter estimates and the covariance matrix from the final PK model, and adopts a multivariate normal distribution as an approximate posterior distribution. Steady‐state C_max_ and AUC were calculated and compared to the reference to obtain the ratio and 95% CI at different covariate values (ie, 10th, 25th, 50th, 75th, and 90th percentiles of observed data for body weight; 10th, 50th, 75th, and 95th percentiles of observed data for age; 10th, 25th, 40th, 60th, 75th, and 90th percentiles of observed data for CrCL). The point estimate and 95% CI were compared to the 0.8‐1.25 region, and covariates with a point estimate and 95% CI outside of that reference range would be considered to have a potentially clinically meaningful effect.

Boxplots were generated to illustrate the relationship between certepetide exposure and CL and renal function. To produce the boxplots, individual empirical Bayes estimates were generated for dataset subjects using the final PK model. Exposure predictions (ie, C_max,ss_ and AUC_ss_) and PK parameter predictions (ie, CL) were generated using the individual empirical Bayes estimates and intrinsic subject covariates present in the dataset. The predicted exposure metrics and PK parameters were then compared against categories of renal function using boxplots.

## Results

### Data Disposition

A summary of subjects and PK samples, stratified by dose, is provided in Table S2. In total, 31 patients with metastatic exocrine pancreatic cancer and 1142 PK observations were included in the population PK analysis. Of the 1142 included observations, 50 (4.4%) were below the limit of quantification and were excluded from the analysis. One outlier data point, defined as an observation with |CWRES| > 5, was identified but was not excluded from the analysis dataset. Eight observations with high predose measurements were excluded from the analysis dataset.

The majority of observations were from subjects receiving a certepetide dose of 1.6 mg/kg (553 observations from 15 subjects) or a dose of 3.2 mg/kg (420 observations from 14 subjects), while fewer observations were from subjects receiving 0.2 mg/kg (69 observations from 5 subjects) or 0.8 mg/kg (50 observations from 4 subjects). Summaries of categorical and baseline continuous covariates, in total and by cohort, are included in Tables S3 and S4, respectively. Of the 31 included subjects, 20 (64.5%) were male and 11 (35.5%) were female; 27 (87.1%) were White, 2 (6.5%) were Black, and 2 (6.5%) were mixed or other race; 19 (61.3%) had normal renal function (CrCL ≥ 90 mL/min), 8 (25.8%) had mild renal impairment (CrCL 60‐89 mL/min), and 4 (12.9%) had moderate renal impairment (CrCL 30‐59 mL/min); 21 (67.7%) had normal hepatic function (aspartate aminotransferase [AST] and bilirubin ≤ upper limit of normal range [ULN]) and 10 (32.3%) had mild hepatic impairment (AST > ULN or ULN < bilirubin ≤ 1.5*ULN). The median (min/max) age, body weight, and CrCL at baseline of all included subjects was 62.1 years (42.6/79.3 years), 73.7 kg (54.0/121 kg), and 96.8 mL/min (48.2/172 mL/min), respectively.

### Exploratory Data Analysis

**Figure 1:**
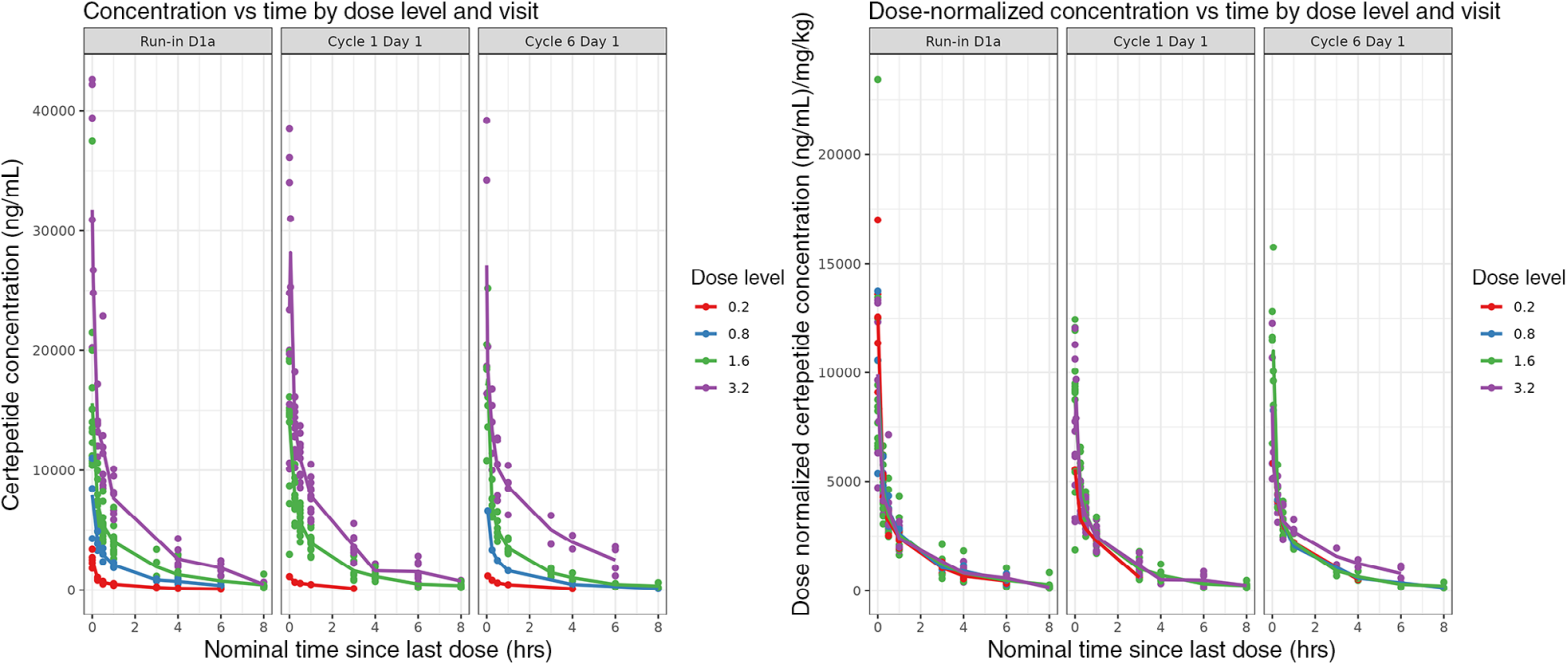
Concentration‐time curves by dose level and visit (raw and dose‐normalized). The solid circles represent observed certepetide concentrations and the lines represent the geometric means of the observed concentrations for each dose level.

Concentration‐time and dose‐normalized concentration‐time curves, stratified by dose level (0.2, 0.8, 1.6, and 3.2 mg/kg), were generated to understand the relationship between observed certepetide concentration and nominal time since the last dose (Figure 1). These plots suggest linear PKs, which informed initial model development.

### Pharmacokinetic Model

Base model development was initiated using a 2‐compartment model with linear elimination, a proportional error model, and interindividual variability included on the following structural model parameters: Vc, CL, Vp. The variance for Q and the variance of an additive part of the residual error model was fixed to 0, as they tended to 0 during the model‐building process. The addition of body weight effect on Vc, CL, and Vp resulted in reduced OFV (ΔOFV = −45.265). OFV was further reduced with the addition of age effect on CL (ΔOFV = −17.07). The effect of sex was then tested on CL and Vc, but a significant effect was not observed (ΔOFV < 10.83), and the covariate was removed in backward elimination. In a final model development step, baseline CrCL was tested on CL. The covariate effect parameter of baseline CrCL on CL replaced the covariate effect parameters of body weight and age on CL because of a strong correlation between CrCL and both age and body weight. This model run resulted in a similar OFV as the model runs with body weight on Vc, CL, Vp, and age on CL (Table S5), but with a more parsimonious structure. Thus, the final PK model selected was a 2‐compartment model with interindividual variability on Vc, CL, Vp, and the following covariate effect parameters: body weight on Vc and Vp, baseline CrCL on CL.

**Figure 2:**
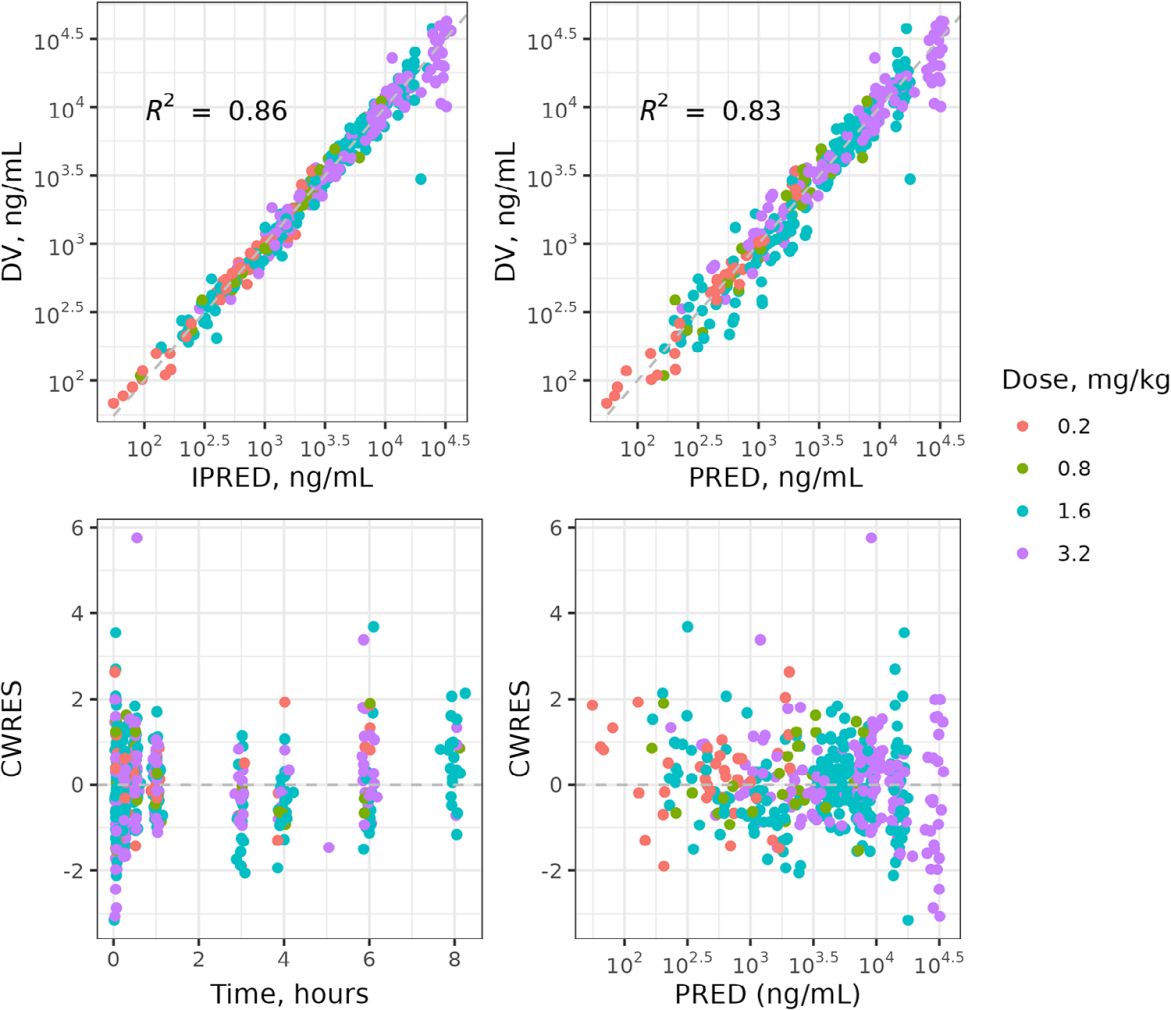
Goodness‐of‐fit plots by dose for the certepetide final PK model. The solid circles in the top 2 plots represent observed certepetide concentrations in comparison to individual and population predicted concentrations. The solid circles in the bottom 2 plots represent weighted residuals in comparison to both population predicted concentration and time. The dashed lines in the top 2 plots represent the line of equality (x = y). The dashed lines in the bottom 2 plots represent the 0 line. DV, observed values; CWRES, conditional weighted residual values; IPRED, individual predictions; PRED, population predictions. PK, pharmacokinetic.

PK parameters for the certepetide final model are presented in Table 1. All PK parameters, as well as interindividual and residual variances, were well estimated with low relative standard error (<50%). Goodness‐of‐fit plots for the certepetide final PK model (Figure 2) demonstrate general alignment between the central tendency of both individual predicted and population predicted concentrations and observed concentrations across dose levels, as well as even distribution of observations around the individual, predicted, and population predicted curves. Plots of CWRES versus time and population‐predicted concentrations do not show any trend and are relatively evenly distributed around 0. Together, these plots suggest the model fits the observed data well.

**Table 1:** Pharmacokinetic Parameters for the Certepetide Final Model

			Estimate	95% CI	Shrinkage (%)
**Structural model parameters**					
Vc (L)	θ_Vc_	Central volume of distribution	5.87	5.23, 6.51	–
CL_NR_ (L/h)	θ_CLNR_	Nonrenal clearance	2.56	1.41, 3.71	–
CL_R_ (L/h)	θ_CLR_	Renal clearance	4.32	3.22, 5.42	–
Vp (L)	θ_Vp_	Peripheral volume of distribution	10.3	9.51, 11.1	–
Q (L/h)	θ_Q_	Intercompartmental clearance	24.9	21.7, 28.2	–
**Covariate effect parameters**					
WT on Vc	θ_WT‐Vc_	Effect of weight on Vc	0.933	0.602, 1.26	–
WT on Vp	θ_WT‐Vp_	Effect of weight on Vp	0.879	0.558, 1.20	–
**Interindividual variance parameters**					
IIV‐Vc	$\omega_{\mathrm{Vc}}^{2}$	Variance for Vc	0.0447 [CV% = 21.4]	0.00996, 0.0795	19.8
IIV‐CL	$\omega_{\mathrm{CL}}^{2}$	Variance for CL	0.0301 [CV% = 17.5]	0.0136, 0.0467	4.99
IIV‐Vp	$\omega_{\mathrm{Vp}}^{2}$	Variance for Vp	0.0209 [CV% = 14.5]	0.00215, 0.0397	25.7
**Residual variance**					
Proportional	$\sigma_{1}^{2}$		0.0393 [CV% = 19.8]	0.0333, 0.0452	7.08

Objective function value = 6334.126; condition number = 27.3; CI = estimate ± 1.96 ∙ SE. CI, confidence interval; CV, coefficient of variation; SE, standard error; IIV, interindividual variance.

**Figure 3:**
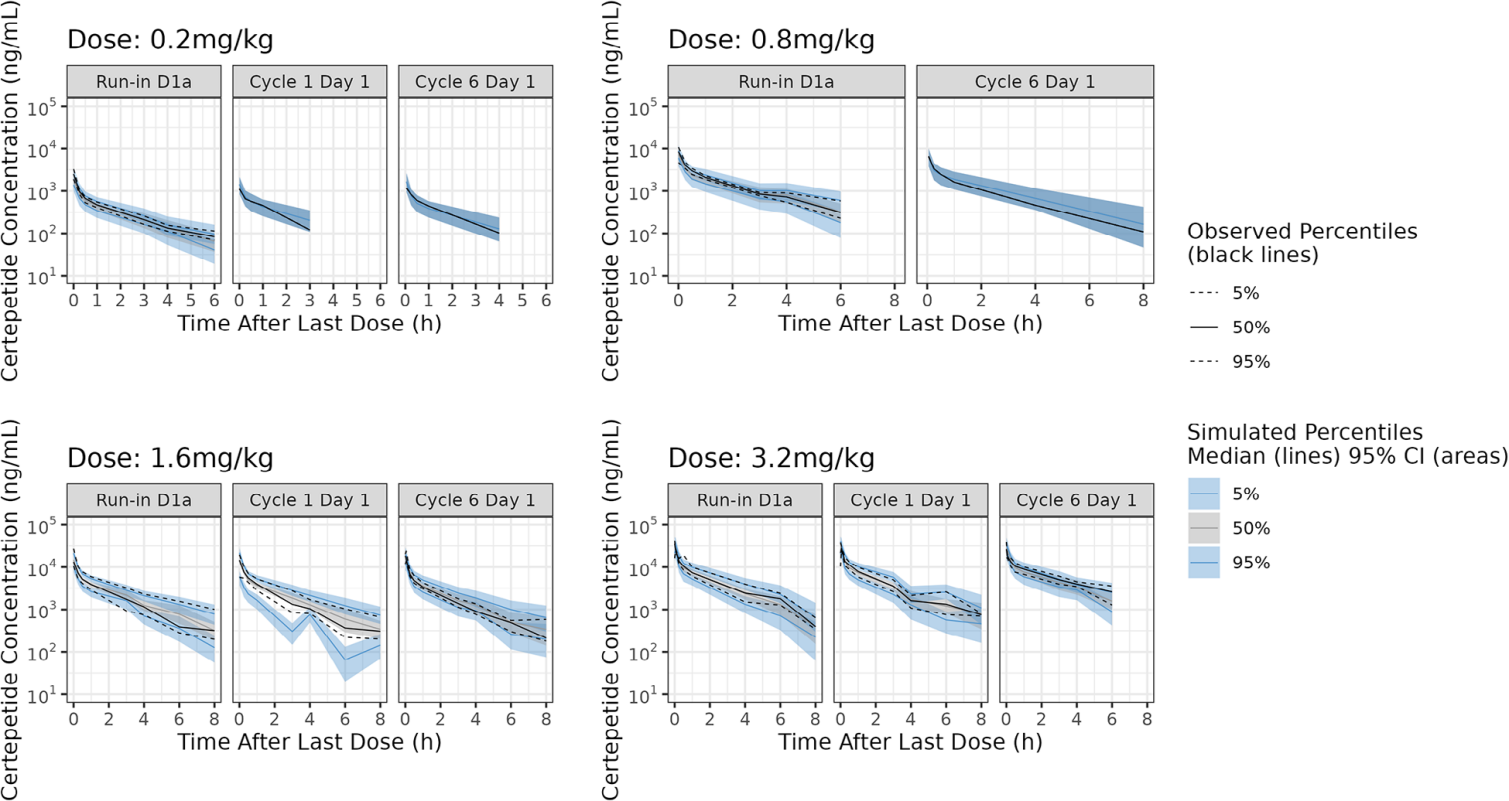
VPC plots by dose for the certepetide final PK model. The lower and upper dashed black lines represent the observed 5th and 95th percentiles. The solid black lines represent the observed 50th percentiles. The lower and upper blue lines and shaded regions represent the median and 95% confidence interval of the simulated 5th and 95th percentiles. The gray lines and shaded regions represent the median and 95% confidence interval of the simulated 50th percentiles. PK, pharmacokinetic; VPC, visual predictive checks.

The ability of the model to simulate the observed data was evaluated using VPCs. VPC plots were generated for the 0.2, 0.8, 1.6, and 3.2 mg/kg dose levels (Figure 3). In all plots, the simulated intervals captured the observed data well overall, suggesting the final PK model is able to adequately predict the observed data for all dose groups.

### Forest Plots

**Figure 4:**
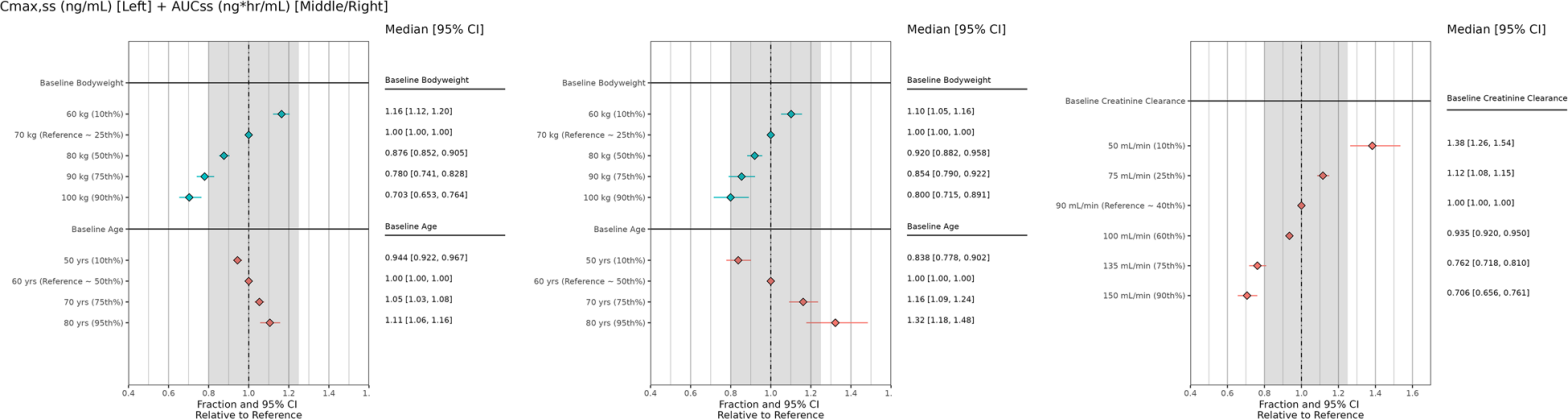
Forest plots of steady‐state C_max_ and AUC by body weight and age and by CrCL. Note: The solid diamonds and horizontal lines represent the median and 95% CI of AUC or C_max_ for each covariate condition. The gray shaded area represents the 0.8‐1.25 reference range. The dashed vertical lines represent 1.00 (ie, no change from reference). AUC, area under the concentration‐time curve; C_max_, maximum concentration; CI, confidence interval; CrCL, creatinine clearance.

Forest plots were used to explore the impact of covariate effects on certepetide exposure, specifically the effects of baseline body weight and age on C_max_ and AUC, as well as baseline CrCL on AUC (Figure 4). A potentially clinically meaningful impact of high body weight (90th per percentile;100 kg) on C_max,ss_ can be observed in Panel A with the median (0.703) falling below the 0.8 to 1.25 reference range. Potentially clinically meaningful impacts of low and high baseline CrCL on AUC_ss_ can be observed in Panel C, with the median 95% confidence intervals of the 10th (50 mL/min) and 90th (150 mL/min) percentiles falling above and below the 0.8‐1.25 reference range (1.38 and 0.706 for the 10th and 90th percentile of CrCL, respectively). These results should be interpreted with caution due to the small sample size of 31 subjects, and further evaluation with a larger sample size is indicated. The medians and 95% confidence intervals of AUC_ss_ for the 25th to 75th percentiles of baseline CrCL fell within or overlapped the 0.8‐1.25 reference range, suggesting no clinically meaningful impact. Since the forest plots compare the fractional change in AUC and C_max_ and the PK of certepetide is linear, similar results can be anticipated across different doses.

### Exposure Predictions

**Figure 5:**
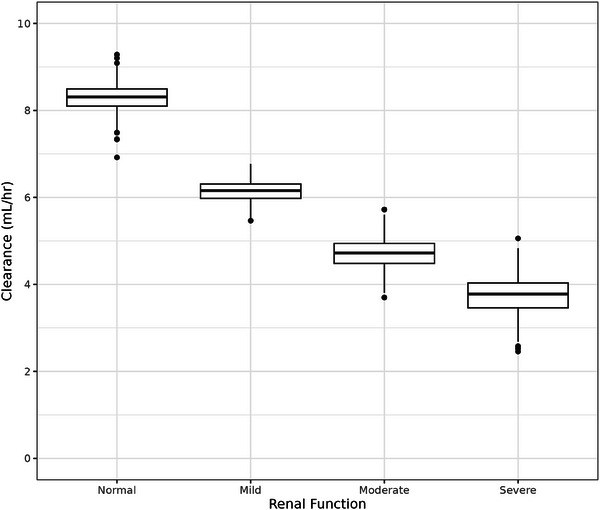
Boxplots of exposure metrics (C_max,ss_ and AUC_ss_) by renal function. The lower and upper bounds of each box represent the 1^st^ quartile (Q1) and 3rd quartile (Q3), respectively. The horizontal line within each box represents the median. The whiskers represent the minimum and maximum values that are within 1.5 IQR below Q1 or above Q3. The solid circles represent outlier data points. Categories of renal function are defined as normal (CrCL ≥ 90 mL/min), mild impairment (CrCL 60‐89 mL/min), moderate impairment (CrCL 30‐59 mL/min), and severe impairment (CrCL < 30 mL/min). AUC_SS_, steady‐state area under the concentration‐time curve; C_MAXSS_, steady‐state maximum concentration.

Boxplots illustrating exposure metrics derived from individual empirical Bayes estimates generated using the final PK model were stratified by categories of renal function to assess the relationship between predicted certepetide exposure and renal function (Figure 5). Categories of renal function were defined as follows: normal CrCL ≥ 90 mL/min, mild impairment CrCL 60‐89 mL/min, moderate impairment CrCL 30‐59 mL/min, and severe impairment CrCL ≤29 mL/min. The lower and upper bounds of each box represent the 1st quartile (Q1) and 3rd quartile (Q3), respectively; the horizontal line within each box represents the median; the whiskers represent the minimum and maximum values that are within the 1.5 interquartile range (IQR) below Q1 or above Q3; the solid circles represent outlier data points. These plots demonstrate a relationship between increasing certepetide exposure with worsening renal function. An inverse relationship was observed in a boxplot illustrating CL derived from the final PK model stratified by categories of renal function (Figure S1), with decreasing CL with worsening renal function.

## Discussion

The population PK model of certepetide was developed using 1142 PK observations from 31 patients with metastatic PDAC receiving certepetide in combination with nab‐paclitaxel and gemcitabine from 1 clinical trial (NCT03517176). A 2‐compartment model with linear elimination, interindividual variability on structural parameters (Vc, CL, Vp), and a proportional residual error structure was found to adequately characterize the PKs of certepetide. Body weight and baseline CrCL were found to have statistically significant effects on volume and CL during model development, as assessed by reduction in OFV, and were included in the final PK model as the following covariate effect parameters: body weight on Vc and Vp; CrCL on CL. Model fit and predictive ability were confirmed through visual evaluation of goodness‐of‐fit and VPC plots.

Of note, the estimated Vc of distribution (5.87 L) was small and close to the average volume of blood for a human adult (ie, 5 L).^27^ The volume of the peripheral compartment was also small (10.3 L), suggesting limited distribution of certepetide. Additionally, non‐renal CL (independent of CrCL; 2.56 L/h) was estimated to be lower than renal CL (dependent on CrCL; 4.32 L/h), which indicates that the kidneys are the primary route of certepetide elimination. Since the parameters for nonrenal CL and renal CL are purely model‐derived rather than experimentally measured, they should be interpreted with caution.

The overall similar predictive performance of the final model (which uses CrCL as a covariate for CL) and the intermediate model (which includes body weight and age as covariates for CL) suggests that, if necessary, dosing can be optimized using either age and body weight or CrCL calculated via the Cockcroft–Gault formula. Both approaches are likely to produce similar outcomes due to the inherent correlations between age, body weight, and CrCL. Since some subjects have CrCL estimates exceeding 120 mL/min, this indicates that the Cockcroft–Gault formula may be biased for this group of patients or patients who exhibit an augmented renal CL. However, this does not diminish the equation's applicability for predictions and dose optimization. The impact of covariate effects on certepetide exposure was further evaluated using forest plots. The plots illustrated potentially clinically meaningful impacts of high body weight (90th percentile, 100 kg) on C_max,ss_ (median 0.708) as well as low (10th percentile, 50 mL/min) and high (90th percentile, 150 mL/min) CrCL on AUC_ss_ (1.38 and 0.706 for the 10th and 90th percentiles of CrCL, respectively), based on medians and 95% confidence intervals outside of the clinical relevance reference range (0.8‐1.25).

The relationship between certepetide exposure (AUC_ss_) and renal function was evaluated using box plots of exposure metrics derived from individual empirical Bayes estimates versus categories of renal function. These plots did demonstrate a relationship between certepetide exposure and renal function, with increasing exposure for increasing degrees of renal impairment. The relationship between CL and renal function was also assessed via boxplots, which demonstrated an inverse relationship between decreasing CL of certepetide with worsening renal function. These trends align with the conclusion that certepetide is primarily eliminated by the kidneys, as indicated by the PK parameter estimates described above.

Renal elimination is common among peptides and peptide conjugates used for the diagnosis and treatment of cancer. A study of ^99m^Tc‐RP527, a diagnostic peptide derived from another peptide (bombesin), found the kidneys to be the primary route of ^99m^Tc‐RP527 CL with rapid elimination of the peptide from the blood.^28^, ^29^ Two additional peptides studied for their tumor diagnostic properties that were also characterized by short CL times and primary elimination through the kidneys are RGD conjugates, [^18^F]Galacto‐RGD, and [^18^F]FPPRGD2.^28^, ^30^, ^31^

One reason peptides may be well suited for renal elimination is their typically small size, characterized by low molecular weight, which allows for glomerular filtration by the kidneys.^32^ It is possible for peptides with molecular weights less than 50 kDa to undergo glomerular filtration.^32^ Peptides with molecular weights less than 10 kDa, such as certepetide (molecular weight 989.1 g/mol, 0.9891 kDa), are able to pass freely through the kidney glomeruli into the urine with the rate of renal CL likely to be similar to the glomerular filtration rate.^33^

Despite the increasing interest in peptides as cancer treatment options,^34^ rapid renal elimination of small peptides such as certepetide can be seen as a limitation.^28^ Rapid renal elimination contributes to a reduced half‐life time of the drug in the blood, which allows less time for the optimal dose of the drug to reach the intended target within the body.^32^, ^35^ In the case of RGD peptides, such as [^18^F]Galacto‐RGD and [^18^F]FPPRGD2^28^, ^30^, ^31^ described above, rapid elimination through the kidneys may reduce the diagnostic capabilities of these compounds for tumors located in the urogenital tract, including tumors that may characterize cancers of the bladder, urethra, prostate, and kidneys.^28^, ^30^ Extending the half‐life of peptides to enhance their therapeutic capabilities is an ongoing area of research.^32^

The findings of this population PK analysis suggest further examination of certepetide efficacy and safety at extremes of body weight and renal function for patients with metastatic PDAC. Importantly, certepetide has a wide margin of safety in animal toxicology studies and a benign safety profile in human clinical studies in which subjects with greater body weight or lesser renal function have not been found to have more AEs.

In the current clinical development, certepetide is dosed based on body weight. However, this analysis suggests that dosing adjusted for CrCL or based on body weight and age would more effectively minimize intersubject variability in drug exposure. The findings from this study may provide clinicians prescribing certepetide for the management of PDAC with a more thorough understanding of the parameters that affect certepetide exposure and elimination, and therefore the tools to optimize individual patient doses.

## Conclusions

Certepetide PK for patients with metastatic PDAC receiving certepetide in combination with nab‐paclitaxel and gemcitabine is characterized by a 2‐compartment model with linear elimination, interindividual variability on structural parameters (Vc of distribution, CL, and Vp of distribution), and a proportional residual error structure. Although certepetide has demonstrated a favorable safety profile both preclinically and clinically, further examination of certepetide safety and efficacy is warranted to account for extremes in renal function impairment and body weight.

## Conflicts of Interest

A.W., A.L., and P.W. are consultants to Lisata Therapeutics, Inc. W.S. and K.B. are employees and stockholders of Lisata Therapeutics, Inc.

## Funding

The clinical study was funded by Cend Therapeutics which was subsequently acquired by Lisata Therapeutics, Inc. The PK modeling which is the subject of this paper was funded by Lisata.

## Supporting information



Supporting Information
