# Supplementary material for: Population Pharmacokinetic Modeling of Certepetide in Human Subjects With Metastatic Pancreatic Ductal Adenocarcinoma
Source: Clin Pharmacol Drug Dev. 2025 Jan 9;14(3):240–51. doi: 10.1002/cpdd.1502 (PMC11905876; doi:10.1002/cpdd.1502)
Supplement: Supplementary file 1 — Supporting Information [file CPDD-14-240-s001.pdf]

Online Supplement

Population Pharmacokinetic Modeling of Certepetide in Human Subjects with Metastatic Pancreatic Ductal Adenocarcinoma

Alex Winning, William K. Sietsema, Kristen K. Buck, Abigail Linsmeier, Pawel Wiczling.

Contents

Table S1. Names and Locations of study sites and responsible Human Research Ethics Committees 2

Table S2. Summary of Subjects and PK Samples by Dose 3

Table S3. Summary of Categorical Covariates by Cohort 4

Table S4. Summary of Baseline Continuous Covariates 5

Table S5. Pharmacokinetic Parameters for Interim Certepetide Model with Covariate Effect Parameters of Body Weight and Age on Clearance 6

Figure S1. Boxplot of Clearance by Renal Function 7

Table S1. Names and Locations of study sites and responsible Human Research Ethics Committees

Name of Study Site	Name of Principal Investigator	Location of Study Site	Responsible HREC	Location of Responsible HREC
**The Queen Elizabeth Hospital**	**Prof Tim Price**	28 Woodville Rd, Woodville South SA 5011 Australia	Central Adelaide Local Health Network	Adelaide Australia
**Alfred Hospital**	**Dr Sanjeev Gill**	55 Commercial Rd, Melbourne VIC 3004 Australia	Sydney Local Health District CRGH Zone	Sydney Australia
**St John of God Hospital**	**Dr. Andrew Dean**	12 Salvado Rd, Subiaco WA 6008 Australia	St John Of God Healthcare Ethics Committee	Subiaco Australia

Abbreviations: HREC = Human Research Ethics Committee

Table S2. Summary of Subjects and PK Samples by Dose

Dose	Number			Percent	
	Subjects	Observations	BLQ	Observations	BLQ
0.2 mg/kg	5	69	5	6.0	0.4
0.8 mg/kg	4	50	2	4.4	0.2
1.6 mg/kg	15	553	22	48.4	1.9
3.2 mg/kg	14	420	21	36.8	1.8
All data	31	1092	50	95.6	4.4

Abbreviations: BLQ = below the limit of quantification

Table S3. Summary of Categorical Covariates by Cohort

	CEND1-001 Study		
	Cohort 1a<br>N = 8	Cohort 1b<br>N = 23	All data<br>N = 31
**Race**			
Caucasian	8 (100.0)	19 (82.6)	27 (87.1)
Mixed/Other	0 (0.0)	2 (8.7)	2 (6.5)
Black	0 (0.0)	2 (8.7)	2 (6.5)
**Sex**			
Male	6 (75.0)	14 (60.9)	20 (64.5)
Female	2 (25.0)	9 (39.1)	11 (35.5)
**Renal Function**			
Normal	--	--	19 (61.3)
Mild	--	--	8 (25.8)
Moderate	--	--	4 (12.9)
**Hepatic Function**			
Normal	--	--	21 (67.7)
Mild	--	--	10 (32.3)

Abbreviations: N = number of records summarized

Notes: Summary is count (percent). Categories of renal function are defined as: normal (CrCL ≥ 90 mL/min), mild impairment (CrCL 60-89 mL/min), and moderate impairment (CrCL 30-59 mL/min). Categories of hepatic function are defined as: normal (aspartate aminotransferase [AST] and bilirubin ≤ upper limit of normal range [ULN]) and mild impairment (AST > ULN or ULN < bilirubin ≤ 1.5*ULN)

Table S4. Summary of Baseline Continuous Covariates

	N	Mean	Median	SD	Min/Max
Age (years)	31	63.8	62.1	10.1	42.6/79.3
Body weight (kg)	31	80.0	73.7	16.8	54.0/121
CrCL (mL/min)	31	102	96.8	36.2	48.2/172

Abbreviations: N = number of records summarized; SD = standard deviation; Min = minimum; Max = maximum; CrCL = creatinine clearance based on Cockcroft-Gault equation.

Table S5. Pharmacokinetic Parameters for Interim Certepetide Model with Covariate Effect Parameters of Body Weight and Age on Clearance

			**Estimate**	**95% CI**	**Shrinkage (%)**
**Structural Model Parameters**					
Vc (L)	θ_{Vc}	Central volume of distribution	5.87	5.19, 6.54	--
CL (L/h)	θ_{CL}	Clearance	7.09	6.53, 7.64	--
Vp (L)	θ_{Vp}	Peripheral volume of distribution	10.3	9.48, 11.1	--
Q (L/h)	θ_{Q}	Intercompartmental clearance	25.0	21.6, 28.4	--
**Covariate Effect Parameters**					
WT on Vc	$\theta_{WT\text{-}Vc}$	Effect of weight on Vc	0.940	0.460, 1.42	--
WT on CL	$\theta_{WT\text{-}CL}$	Effect of weight on CL	0.630	0.306, 0.954	--
WT on Vp	$\theta_{WT\text{-}Vp}$	Effect of weight on Vp	0.885	0.520, 1.25	--
Age on CL	$\theta_{Age\text{-}CL}$	Effect of age on CL	-0.986	-1.39, -0.580	--
**Inter-individual Variance Parameters**					
IIV-Vc	ω_{Vc}^2	Variance for Vc	0.0450 [CV% = 21.5]	0.0102, 0.0798	19.8
IIV-CL	ω_{CL}^2	Variance for CL	0.0288 [CV% = 17.1]	0.0128, 0.0447	5.16
IIV-Vp	ω_{Vp}^2	Variance for Vp	0.0195 [CV% = 14.0]	0.00137, 0.0376	26.9
**Residual Variance**					
Proportional	σ_1^2		0.0394 [CV% = 19.8]	0.0334, 0.0454	7.01

Abbreviations: CI = confidence interval; CV = coefficient of variation; SE = standard error

Note: Objective Function Value = 6333.066; Condition Number = 6.55

Note: CI = estimate ± 1.96 · SE

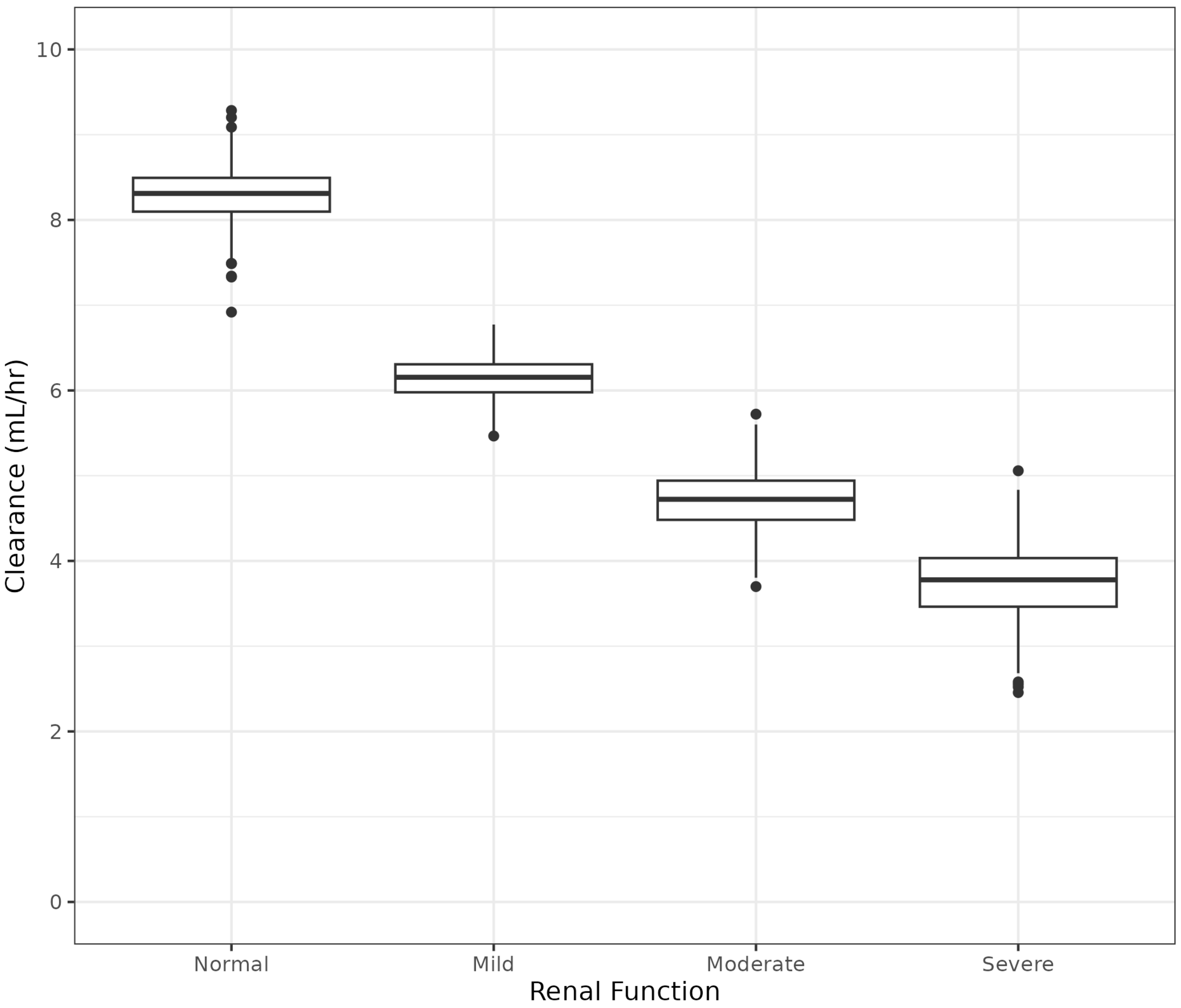

Note: Results for severe renal impairment should be interpreted with caution as these results are based on extrapolation outside of the range of observed creatinine clearance in the analysis dataset.

Note: The lower and upper bounds of each box represent the 1st quartile (Q1) and 3rd quartile (Q3), respectively. The horizontal line within each box represents the median. The whiskers represent the minimum and maximum values that are within 1.5 IQR below Q1 or above Q3. The solid circles represent outlier datapoints.

Note: Categories of renal function are defined as: normal (CrCL ≥ 90 mL/min), mild impairment (CrCL 60-89 mL/min), moderate impairment (CrCL 30-59 mL/min), and severe impairment (CrCL < 30 mL/min).

Figure S1. Boxplot of Clearance by Renal Function
